# ESPO AND BIOLOGICAL SCIENCES SECTION SYMPOSIUM: METABOLIC REGULATION OF AGING

**DOI:** 10.1093/geroni/igae098.1868

**Published:** 2024-12-31

**Authors:** Bhaswati Ghosh

**Affiliations:** Chair

## Abstract

We are excited to announce that the 2024 GSA Annual Scientific Meeting will hold the Emerging Scholar and Professional Organization (ESPO) section of BioSci on November 16, 2024, 12:00 pm – 1:30pm (Room 4C-3). This session continues to be the highlight of the program, recognizing the great work done by the graduate students in the field of biology of aging. The BioSci ESPO section will comprise four talks covering diversifying topics including proteostasis, mitochondrial bioenergetics, lipidomics and transcriptional changes during aging. We will hear from Ziying Xu from Barshop Institute Department of Medicine talking about “Sex-Specific Alterations in Circulating Lipidome During Mouse Aging”; Sidharth Madhavan at Buck institute for research on aging about “Beta-Hydroxybutyrate Is a Metabolic Regulator of Proteostasis in the Aging Brain”; Stephanie Heimier from UC San Diego will give us insights on “Serum Factors Drive Differences in Mitochondrial Bioenergetics Associated with Human Aging”; and Ruben De Man from Yale University school of medicine will present on “Single-Cell Analysis of Somatic Mutations in Human Lung Reveals Association with Transcriptional Changes in Aging”. The BioSci ESPO session at the GSA Annual Scientific Meeting is designed to showcase the academic excellence of great researchers in the field of aging. We are excited to see you all in Seattle!

